# Mesiodistal Measurements for Dental Implant Planning: A Prospective Clinical Study of Linear Measurements on Cone-Beam Computed Tomography Images in Comparison with Caliper-Based Measurements on Plaster Casts

**DOI:** 10.3390/dj10090169

**Published:** 2022-09-07

**Authors:** Lydia Vazquez, Ramona Buser, Jean-Pierre Carrel

**Affiliations:** 1 Department of Orofacial Rehabilitation, University Clinics of Dental Medicine, University of Geneva, Rue Michel-Servet 1, 1211 Geneva, Switzerland; 2 Department of Reconstructive Dentistry and Gerodontology, School of Dental Medicine, University of Bern, Freiburgstrasse 7, 3010 Bern, Switzerland; 3 Department of Preventive Dental Medicine and Primary Dental Care, University Clinics of Dental Medicine, University of Geneva, Rue Michel-Servet 1, 1211 Geneva, Switzerland

**Keywords:** CBCT imaging, clinical trials, dental implant, linear measurements, radiology

## Abstract

Althouh cone beam computed tomography (CBCT) is popular for dental implant planning, the horizontal mesiodistal space of the edentulous ridge is still conventionally measured with a handheld sliding caliper in the oral cavity or on a plaster cast. For clinical application in implant planning, our aim was to evaluate the accuracy of CBCT horizontal mesiodistal linear measurements in comparison with conventionally obtained direct measurements on plaster casts. Postoperative CBCT acquisitions and plaster casts of 27 patients with adjacent posterior mandibular implants were analyzed in a prospective clinical study. On CBCT images, two observers assessed the inter-implant distances on axial and sagittal views. On plaster casts, the inter-implant distances were measured with a digital caliper. CBCT measurements on axial and sagittal views were, on average, 0.2 mm larger than measurements on plaster casts. Correlation was perfect between measurements of the same inter-implant distance performed by the observers on CBCT images and on plaster casts. When compared with conventionally obtained direct measurements on plaster casts, CBCT views slightly overestimated (mean 0.2 mm) the horizontal mesiodistal measurements between two implants as reference points. CBCT imaging is sufficiently accurate to evaluate mesiodistal distances on axial and sagittal views for dental implant planning in clinical practice.

## 1. Introduction

Correct management of the edentulous space is essential for successful dental implant treatment. The dimensions of the edentulous ridge are evaluated to determine the distribution, size and number of implants. To allow for maintenance and to avoid the loss of marginal bone, adequate space from the adjacent tooth to the center of the implant or between two adjacent implant shoulders is necessary when planning multiple adjacent implants [1,2]. In partially edentulous patients, the horizontal mesiodistal (MD) space is the length of the gap measured at bone level between the necks of the teeth on each side of an edentulous ridge. Although Cone-Beam Computed Tomography (CBCT) is commonly used before dental implant surgery, the MD space is still conventionally measured with a handheld sliding caliper in the oral cavity or on a plaster cast. The exact length of the edentulous ridge can be difficult to measure with a caliper in the oral cavity before surgery; the caliper can be positioned incorrectly (such as when a tooth is mesially tilted or when the patient has a reduced mouth opening), influencing the MD measurement of the edentulous ridge.

Many implant-related CBCT publications are presurgical CBCT studies [3] focused on bone height and the width of the edentulous ridge, with the issue of measurement accuracy on CBCT images receiving considerable attention over the years. Phantom heads [4]; dry skulls and human cadavers [2,5,6,7]; porcine mandibles [8,9]; beagle dog mandibles [10]; virtual models [11,12]; and reference objects, such as implants [13,14], have been used to assess the accuracy of linear measurements on CBCT views. Some authors have stated that CBCT views enable accurate measurements [6], whereas other authors report underestimated [5] or overestimated CBCT measurements [8]. A systematic review identified 22 studies that have assessed the accuracy of CBCT linear measurements [15]. Only two studies included in that review were clinical studies. With respect to bone height and width measurements, the authors found “no clear trend as to whether CBCT measurements are consistently under or overestimated” [15]. Publications reporting on MD bone dimensions measured on CBCT images for implantology purposes have involved the study of dry skulls and dry human mandibles [2,7]. To the best of our knowledge, horizontal length measurements at the crestal level of patients on CBCT images and on plaster casts have not been compared in the literature.

For clinical application in implant planning, our aim was to evaluate the accuracy of horizontal MD linear measurements on CBCT images in comparison with conventionally obtained direct measurements on plaster casts. Another aim was to evaluate, in real clinical settings, whether the length of the edentulous space affects the measurement accuracy of CBCT. We hypothesized that CBCT imaging is sufficiently accurate for horizontal MD measurements for implant planning and that clear landmarks fixed on the edentulous ridge, both for CBCT measurements and for conventionally obtained reference measurements on plaster casts, would allow CBCT horizontal linear measurements to be assessed for accuracy. We believe that the settings of this clinical study allow for a more reliable evaluation of the accuracy of horizontal MD measurements on CBCT views than if horizontal distances were measured between the necks of the teeth on either side of the edentulous ridge.

## 2. Materials and Methods

### 2.1. Study Design

This study was designed as a prospective single-center clinical study to evaluate the accuracy of horizontal linear measurements on CBCT views in comparison with conventionally obtained direct measurements on plaster casts (gold standard). The present study implements the STROBE guidelines and was conducted in accordance with the Declaration of Helsinki and good clinical practice guidelines. The study protocol was approved by the Ethics Committee for Clinical Research of the Geneva Medical Association, Switzerland (protocol 11-10).

### 2.2. Patient Screening and Selection

Measurements on CBCT views and on plaster casts were evaluated in this clinical trial. A sample size of 24 patients was required to assess the mean error with a 95% confidence interval of ±0.10 mm around the mean. In anticipation of potentially unusable data and patients lost to follow-up, 30 patients were enrolled.

Study participants were recruited from patient referrals for implant surgery at the University Clinics of Dental Medicine (University of Geneva, Geneva, Switzerland). At an initial preoperative implant evaluation, the study protocol was thoroughly explained to each participant. Each participant received an information handout detailing the full study protocol and signed an informed consent form. The preoperative evaluation included a clinical evaluation of the available MD space (to ensure sufficient space for two implants) and 2D imaging implant planning. The standardized one-stage surgical procedure and follow-up are described in a previous prospective study [16]. Surgical procedures were performed by oral surgeons with a range of experience. The study design replaced postoperative 2D imaging (periapical or panoramic X-ray) with CBCT imaging to allow for various measurements linked to dental implants inserted without surgical guides in the posterior mandible. Implant-supported rehabilitations were fabricated 6 to 8 weeks after surgery by the Department of Fixed Prosthodontics and Biomaterials at the University Clinics of Dental Medicine.

Participant inclusion criteria:

- Partially dentate patients 20 years of age and older;
- Patients requiring premolar and/or mandibular implants inserted in a one-stage surgery;
- Patients requiring two or more adjacent implants with the following characteristics: RN 8 mm length SP (Tissue Level, Institut Straumann AG, Basel, Switzerland) and a 1.5 mm height healing cap;
- Patients willing to undergo an immediate postoperative CBCT examination; and
- Patients willing to undergo conventional impressions of the osseointegrated adjacent implants 6 to 8 weeks after surgery and shortly before removal of healing caps for implant-supported crown or bridge restauration.
- Participant exclusion criteria:
- Medical history of bisphosphonate intake, uncontrolled diabetes, head or neck radiation therapy or metabolic bone disease;
- Clinical or radiological signs of active or untreated periodontal disease;
- Pregnancy or breastfeeding; and
- Patients requiring bone augmentation prior or concomitant to implant placement.

A grant covered the costs of conventional impressions, CBCT examinations and analyses. Patient recruitment was hindered by the restrictive inclusion criteria with respect to implant diameter and length (4.1 mm standard diameter and 8 mm length) and by the cost of implant treatment. The ethics committee approved the following modifications to the original protocol: patients requiring two or more adjacent posterior mandibular implants of any diameter and length from the Straumann implant brand, with or without bone augmentation technique prior or concomitant to implant placement. A supplementary grant covered the material costs of 36 dental implants, and 18 more patients were recruited.

### 2.3. CBCT Acquisitions and Measurements

**Figure 1:**
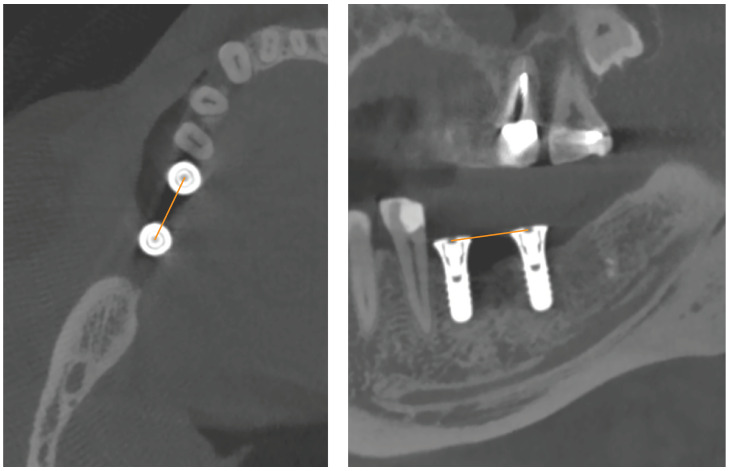
Horizontal linear measurements from the center of one implant healing cap to another (i.e., inter-implant distance) on CBCT axial and sagittal views.

Postoperative CBCT imaging replaced postoperative intraoral or panoramic X-ray in this prospective study. After implant surgery, study participants were sent to the Geneva University Hospitals (Switzerland) for a CBCT (NewTom VGi, Quantitative Radiology, Verona, Italy). Images were acquired using high-resolution mode (voxel size: 0.15 mm; field of view: 12 × 8 cm), and exposure parameters were set to 110 kVp, 3–11 mA. On NNT viewer, primary reconstructions were made with an isotropic voxel (pixel size: 0.15 mm; slice thickness: 0.15 mm). The acquisition data were saved on the PACS Digital Imaging and Communications in Medicine (DICOM) server (512 × 512 matrix) and exported to a compact disc. Two observers (J.P.C. and L.V.: observers 1 and 2, respectively), experts in oral surgery and radiology, took the measurements twice; sessions occurred at least 2 weeks apart. Axial, sagittal in multiplanar reconstruction mode. On the axial view, the cross-reference lines were aligned with the center line between the implant healing caps to display the two implants side by side on the sagittal oblique (hereafter sagittal) view. On the coronal view, the cross-reference lines were oriented to view the center of both healing caps on the sagittal views. On the CBCT axial and sagittal views (Figure 1), the inter-implant distance (i.e., the distance from the center of one implant healing cap to another) was measured with OsiriX DICOM viewer (Pixmeo, Geneva, Switzerland) installed on an iMac OS X (Apple Inc., Cupertino, CA, USA) independent workstation with a 2560 × 1440 resolution LCD screen.

### 2.4. Plaster Casts Measurements

Prior to implant restoration 6 to 8 weeks after surgery, a conventional impression was taken with a vinyl polysiloxane impression material (Express, 3M ESPE, Seefeld, Germany) and poured with dental stone. The measurements on plaster casts served as the gold-standard reference. Two observers (L.V. and R.B.: observers 2 and 3, respectively) measured each inter-implant distance twice with a digital caliper (FINO GmbH, Bad Bocklet, Germany, precision: 0.01 mm). Sessions occurred at least 3 weeks apart. The type of implant-supported restoration (single crown vs. bridge), the type of tooth replaced by an implant (premolar vs. molar) and its localization (right vs. left) were reported in a spreadsheet.

### 2.5. Statistical Analysis

CBCT data of patients lost to follow-up were excluded. For the remaining 27 patients, 324 inter-implant measurements on CBCT images and plaster casts were analyzed. The mean age was described for each gender. The mean, median and interquartile ranges (25% to 75%) were described for the inter-implant spaces. We calculated the agreement between observer measurements (observer 1 vs. observer 2 and observer 2 vs. observer 3) for each method (CBCT axial view, CBCT sagittal view and plaster cast) by calculating the intra-class correlation coefficient (ICC). Different options for calculating the ICC generate different results when applied to the same data [17]. We used a two-way random effects model (“icc” command in Stata) and specified both the rater variable ‘observer’ and the target variable ‘implant’. We reported the individual absolute agreement ICCs, which correspond to ICC(2,1) [17]. For observer 2, we calculated the agreement between measurement methods (CBCT axial vs. CBCT sagittal, CBCT axial vs. plaster cast and CBCT sagittal vs. plaster cast) by calculating the ICC. We used a two-way random effects model (“icc” command in Stata) and specified both the ‘method’ variable (CBCT axial view, CBCT sagittal view and plaster cast) and the target variable ‘implant’. We reported the individual absolute agreement ICCs, which correspond to ICC(2,1) [17]. We arbitrarily chose first session measurements to compute ICCs.

**Figure 2:**
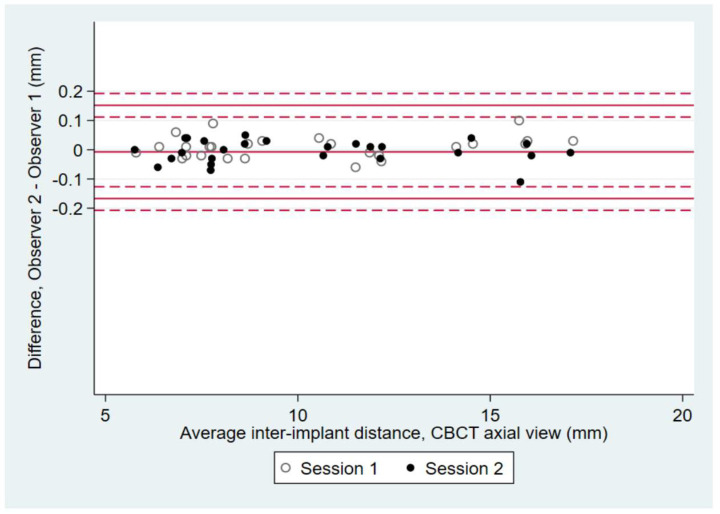

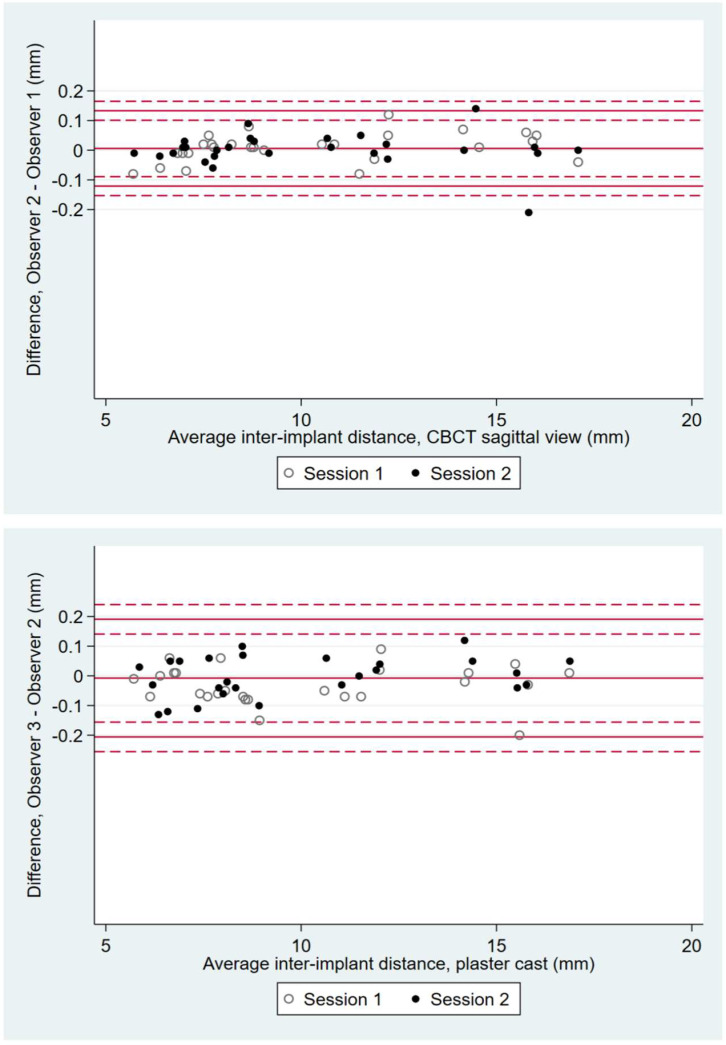
Bland–Altman diagrams show inter-observer reproducibility coefficients and 95% limits of agreement for observers 1 and 2 on CBCT axial and sagittal views, and for observers 2 and 3 on plaster casts. The inter-implant distances ranged from 5.6 mm to 17.1 mm. The representation of the mean difference (middle solid line), the confidence interval limits for mean (upper and lower solid lines) and the 95% agreement limits (dotted lines) are shown on the diagrams.

**Figure 3:**
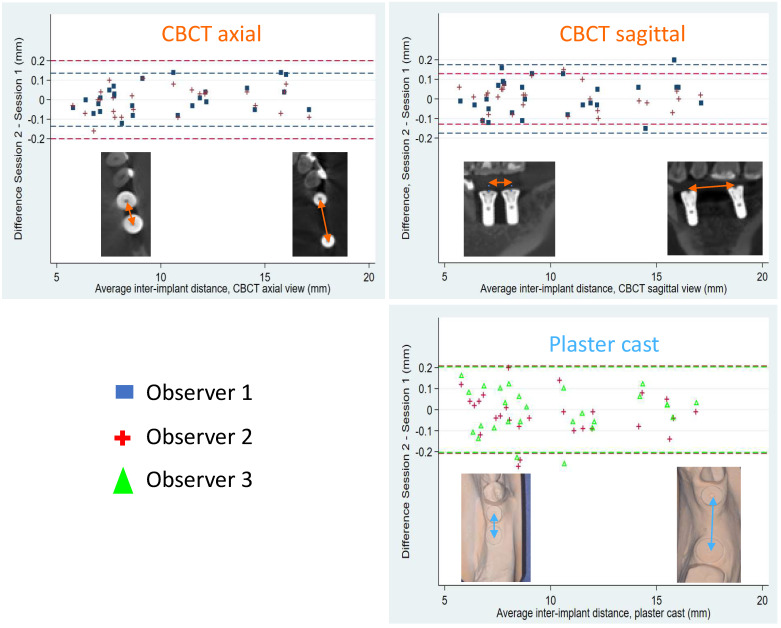
Bland–Altman diagrams show intra-observer repeatability coefficients and 95% limits of agreement for observers 1 and 2 on CBCT views and for observers 2 and 3 on plaster casts. Inter-implant distances ranged from 5.6 mm to 17.1 mm (photo insets). The representation of the mean difference (middle solid line), the confidence interval limits for mean (upper and lower solid lines) and the 95% agreement limits (dotted lines) are shown on the diagrams.

Inter-observer reproducibility was studied for each measurement method (CBCT axial and sagittal views, as well as plaster cast measurement); limits of agreement were calculated with their 95% confidence intervals and reported on a Bland–Altman diagram (Figure 2). Intra-observer repeatability was analyzed by calculating the repeatability coefficients after performing a one-way analysis of variance, with implant as the factor, to estimate the within-subject standard deviation of the measurement. Limits of agreement were calculated with their 95% confidence intervals and reported on a Bland–Altman diagram (Figure 3). For CBCT measurements, a one-way analysis of variance was performed for observers 1 and 2; for plaster cast measurements, one-way ANOVA was performed for observers 2 and 3.

**Figure 4:**
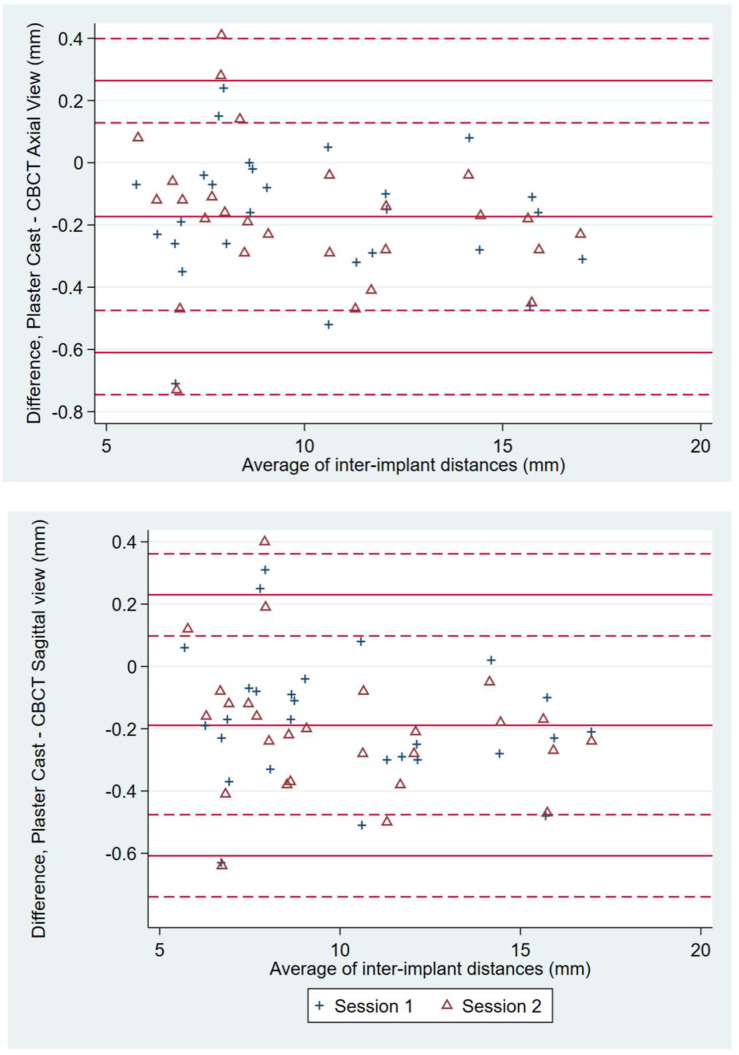
For observer 2, the effect of each method (plaster cast vs. CBCT axial view and plaster cast vs. CBCT sagittal view) on the average measurement was examined using a linear regression mixed model. Methods and observers were considered fixed effects.

For observer 2, agreement between measurements on CBCT axial views (and respective sagittal views) and plaster casts were reported on Bland–Altman diagrams (Figure 4). Limits of agreement were calculated with their 95% confidence intervals, taking into account the repeated-measurements structure of our data. The effect of each method (CBCT axial views vs. plaster cast and CBCT sagittal views vs. plaster cast) on the average measurement was analyzed with a linear regression mixed model of the inter-implant spaces. Methods and observers were considered fixed effects. Random intercepts at the implant level were considered. Data were analyzed with Stata (StataCorp. 2017. Stata Statistical Software: Release 15. College Station, TX, USA: StataCorp LLC).

## 3. Results

### 3.1. Participants

A total of 32 participants met the eligibility requirements, received a copy of the study protocol and signed the informed consent form at the initial preoperative implant evaluation. One patient withdrew from the study, preferring a single-implant treatment; a second patient chose a partial removable rehabilitation. Thus, 30 participants enrolled in the study underwent CBCT imaging after implant surgery. Three of thirty participants were lost to follow-up (they did not return for the conventional impression session). Of 27 remaining participants, 19 were women (mean age 58.7 ± 8.6 years), and 8 were men (mean age 66.2 ± 8.6 years).

### 3.2. Inter-Implant Distances

All 54 (100%) dental implants were osseointegrated, and 36/54 dental implants replaced missing molars. A total of 38 adjacent single-crown restorations and 8 implant-supported bridges were performed. A total of 27 inter-implant spaces served as fixed measured objects; 19/27 inter-implant spaces were in the posterior left mandible. The shortest inter-implant distance measured 5.76 mm, and the longest measured 17.17 mm. On CBCT axial views, the mean inter-implant distance was 10.24 ± 3.4 mm (median, 8.72 mm). On sagittal views, the mean inter-implant distance was 10.26 ± 3.4 mm (median, 8.72 mm). On plaster casts, the mean inter-implant distance was 10.06 ± 3.4 mm (median, 8.60 mm).

### 3.3. ICC

ICCs estimate correlations between individual measurements and average measurements made on the same target. For CBCT axial views, the ICC between observers 1 and 2 was almost 1.0, indicating perfect correlation between measurements of the same inter-implant distance performed by the two observers. This was also the case for CBCT sagittal views. For plaster casts, the ICC between observers 2 and 3 was almost 1.0, indicating perfect correlation between measurements of the same inter-implant distance performed by the two observers (Table 1).

### 3.4. Inter-Observer Reproducibility

The inter-observer reproducibility for CBCT measurements is acceptable (Figure 2). On axial views, the mean difference between observers 1 and 2 was −0.01 mm (−0.17 and 0.15 mm lower and upper limits of agreement, respectively). On sagittal views, the mean difference between observers 1 and 2 was −0.01 mm (−0.12 and 0.13 mm lower and upper limits of agreement, respectively). On plaster casts, the mean difference between observers 2 and 3 was −0.01 mm (−0.21 and 0.20 mm lower and upper limits of agreement, respectively). Limits of agreement were narrower on CBCT images than on plaster casts, suggesting that inter-observer reproducibility was better for CBCT images than for plaster casts.

### 3.5. Intra-Observer Repeatability Coefficients

Overall, intra-observer repeatability coefficients were lower for CBCT images (observer 1: 0.14 and 0.18 mm for axial and sagittal views, respectively; observer 2: 0.20 and 0.13 mm for axial and sagittal views, respectively) than for plaster casts (0.20 mm and 0.21 mm for observers 2 and 3, respectively) (Figure 3). Thus, intra-observer repeatability coefficients were inferior or equal to 0.2 mm (for measurements on CBCT images or on plaster casts), which is acceptable. The threshold for clinical significance was preset at 0.4 mm.

For observer 2, who performed measurements on CBCT images and on plaster casts, the mean difference between measurements on CBCT axial views and measurements on plaster casts was −0.17 mm (−0.61 mm and 0.26 mm lower and upper limits of agreement, respectively) (Figure 4). The mean difference between measurements on CBCT sagittal views and measurements on plaster casts was −0.19 mm (−0.61 mm and 0.23 mm lower and upper limits of agreement, respectively). Thus, measurements on CBCT sagittal views were, on average, 0.19 mm larger than those on plaster casts (*p* < 0.001) (Table 2). However, measurements on CBCT images were as much as 0.6 mm larger than measurements on plaster casts. After adjusting for the observer, the linear regression mixed model showed that inter-implant distances were significantly longer on CBCT images than on plaster casts (*p* < 0.001 for both axial and sagittal views compared with plaster casts). When measuring MD spaces, we found no significant differences between axial and sagittal views on CBCT images (*p* = 0.551).

## 4. Discussion

Authors of in vivo and in vitro studies have reported differences in the *height* and *width* of bone measurements on CBCT images when compared with various standard reference measurements [5,6,15].

We are the first to report on the use of two dental implants as landmarks fixed in the mandibular alveolar ridge of patients to measure the length of horizontal distances on CBCT views in real clinical settings. We report excellent ICCs between observers 1 and 2 for CBCT measurements on axial and sagittal views and perfect ICCs (almost 1.0) between measurements on CBCT views and on plaster casts. Inter-observer reproducibility was better for measurements on CBCT images than for measurements on plaster casts (i.e., narrower limits of agreement for CBCT images than for plaster casts). Overevaluation did not differ significantly between axial and sagittal measurements. The limits of agreement were in the range of −0.17 mm to 0.15 mm on axial views and in the range of −0.12 mm to 0.13 mm on sagittal views.

Inter-implant distances measured on CBCT images had better intra-observer repeatability coefficients than inter-implant distances measured on plaster casts, ranging from 0.13 mm to 0.20 mm, which is acceptable and met our threshold for clinical significance preset at 0.4 mm. MD dimensions of impacted tooth germs measured directly with a sliding caliper after osteotomy were compared with MD tooth dimensions on CBCT-3D-generated models; the teeth were 0.26 mm smaller on CBCT views than the same teeth measured with a caliper [9]. In contrast, other authors have shown that MD dimensions of teeth on CBCT views were wider than clinical measurements [18]. Authors of another orthodontic study assessed the MD dimensions of posterior teeth on CBCT views and on plaster casts [19]. They set a clinically relevant threshold of 0.5 mm and reported mean intra-observer differences of repeated measurements of 0.12 ± 0.10 mm for mandibular premolars and 0.10 mm ± 0.12 mm for mandibular molars. In the same report, the mean differences between MD measurements of teeth on CBCT views and those on plaster casts were 0.32 ± 0.30 mm for mandibular premolars and 0.31 ± 0.22 mm for mandibular molars [19].

For observer 2 in the present study, measurements on axial views were, on average, 0.17 mm larger than measurements on plaster casts; measurements on sagittal views were, on average, 0.19 mm larger than those on plaster casts (*p* < 0.001). The mean differences and the limits of agreement suggest discrepancies between the two measurement methods. Overestimated dimensions of the edentulous ridge can affect the planning of implant distribution along the crest, resulting in overcrowded implants. We believe that the maximum 0.6 mm overestimation reported in the present study when measuring horizontal inter-implant distances on CBCT views is not clinically significant with respect to implant planning, particularly when assessing long edentulous MD spaces. Authors assessing bone length measurements on CBCT images of dry human mandibles have reported a >1.5 mm divergence when the mandibles were tilted in various planes (5- to 10-degree angulation), whereas the mean error value was 0.53 ± 0.08 mm when measuring bone length with ideal mandible positioning [2]. They found that horizontal bone length measurements were more impacted by rotation of the mandible than bone height and width measurements. They also stated that improper head positioning can lead to variations when measuring bone dimensions for implant purposes in multiplanar reconstruction mode. The results of our clinical study, showing minimal differences between measurements on plaster casts (gold standard) and measurements on CBCT views, tend to confirm that the patients were ideally positioned during CBCT acquisition and that the reformatted image plane for the on-screen measurements was also correct.

Some authors have stated that on-screen measurements with a computer mouse are more valid than conventional caliper measurements on plaster casts because “there is no physical barrier of the caliper dictating placement of measurement points” [20]. When measuring an edentulous space mesiodistally on a plaster cast with a caliper, changes in the position of the caliper tips can lead to random errors [11]. Changes in the position of the caliper tips were less likely to occur in the present clinical study, as the centers of the healing caps, which served as two landmark measurements, were visually unambiguous and easily identifiable center points on the plaster casts. We found that intra-observer repeatability coefficients for measurements from the center of one implant healing cap to another on plaster casts were around 0.2 mm, which is acceptable and in accordance with studies reporting on teeth dimensions. When measuring the MD dimensions of teeth on plaster casts with a caliper, intra-observer differences ranged from 0.05 ± 0.02 mm to 0.24 ± 0.14 mm [19].

Another aim of the present study was to examine whether the length of the measured edentulous space affects the accuracy of CBCT horizontal linear measurements. One requirement for inclusion in this prospective study was patients requiring two adjacent implants to replace two or more missing teeth in the posterior mandible. The edentulous ridge was longer for 8 patients requiring an implant-supported bridge restoration than for 19 patients requiring 2 single crowns on implants. Our results suggest that the length of the inter-implant space (whether short or long) did not affect the accuracy of CBCT horizontal linear measurements. We found no human clinical studies that compare shorter and longer horizontal measurements for dental implant planning. In an animal study of ex-vivo porcine mandibles, CBCT measurements were compared with digital caliper measurements; the mean differences were 0.52 ± 0.36 mm (ranging from 0.01 to 1.42 mm) for the shorter distances and 0.37 ± 0.29 mm (ranging from 0 to 1.39 mm) for the longer distances [8]. The same authors reported that CBCT measurements underestimated 76% of shorter and 51% of longer distances as compared with direct measurements.

The methodology employed in this clinical study is sound, with accessible landmarks to measure MD distances on CBCT views and on plaster casts. One limitation is the small number of inter-implant spaces located exclusively in the posterior mandible. Other potential limitations include artefacts related to the presence of titanium implants and the high-resolution CBCT acquisitions. The FOV size and acquisition mode are the settings utilized in our earlier preclinical study [14]. In the present study, 3/27 CBCT acquisitions presented lower quality images. Visible motion artefacts, probably related to the high-resolution protocol requiring longer scan times, making images susceptible to patient movement during acquisition [21], could have contributed to the maximum 0.6 mm overestimation on CBCT views. Metal and titanium implants produce streak artefacts and beam-hardening artefacts [14,21]. We carefully selected the landmarks (the center of each dental implant) with the on-screen cursor (guided by a computer mouse). We encountered no difficulty identifying the exact center point on each implant’s healing cap. However, implants appeared slightly oval-shaped on axial views, and dark streaks appeared between implants on sagittal sections. CBCT measurements of dental implants of various brands inserted in the jaws of patients were compared with the real implant dimensions listed by the manufacturers [13]. Findings were unrelated to implant material (titanium or titanium-zirconium); horizontal dimensions on CBCT cross-sectional and sagittal views were 0.11 mm to 0.37 mm (3.34%) larger than the real implant size [13]. For Straumann dental implants (the same brand used in the present study), horizontal measurements of the implant diameter on CBCT views were 0.11 mm larger than the real implant size. The authors, who did not distinguish between cross-sectional and sagittal horizontal measurements, concluded that implants affected neither the quality of CBCT views nor the measurement precision [13]. Our clinical findings confirm that the presence of implants did not affect inter-implant measurements on CBCT views. However, in comparison with the reference measurements conventionally obtained with a digital caliper on plaster casts, horizontal linear measurements were slightly overestimated on CBCT views (mean 0.2 mm).

The OsiriX image-viewer used for CBCT measurements in the present study might have affected the results. OsiriX viewer has been assessed for measurement accuracy and reliability on computed tomography images of pig femurs [22]. The authors reported that OsiriX viewer was very reliable for measurement purposes (less than 0.1 mm difference between real and OsiriX measurements). In an in vitro preclinical study of vertical measurements on CBCT views acquired on the NewTom VGi CBCT machine used in the present study, we obtained slightly more accurate measurements on OsiriX viewer than on the NewTom viewer (Quantitative Radiology, Verona, Italy) [14]. The mean measurement differences were −0.01 ± 0.03 mm on cross-sectional and 0.03 ± 0.04 mm on sagittal views. In that in vitro study, metal artefacts did not influence the accuracy of measured implant lengths; hence, an equivalent accuracy with OsiriX viewer can be expected in the present clinical study when measuring MD distances on axial and sagittal views. To further analyze the accuracy of CBCT horizontal linear measurements along the alveolar ridge, a future clinical study could include a wider pool of patients with inter-implant spaces in the anterior and posterior segments of upper and lower jaws, with CBCT machines from different manufacturers for image acquisition.

## 5. Conclusions

When compared with conventionally obtained direct measurements on plaster casts (gold standard), CBCT views slightly overestimated (mean, 0.2 mm) horizontal MD measurements between two implants used as reference points. CBCT imaging is sufficiently accurate to evaluate MD distances on axial and sagittal views for dental implant planning in clinical practice.

## Figures and Tables

**Table 1:** Intra-class correlation coefficients (ICC) show the correlation between observers and methods.

Inter-observer ICC	CBCT axial views (obs 1 vs. 2)	0.9999330
	CBCT sagittal views (obs 1 vs. 2)	0.9998992
	Plaster casts (obs 2 vs. 3)	0.9994949
Inter-method ICC (for observer 2)	CBCT axial vs. sagittal views (obs 2)	0.9997868
	CBCT axial views vs. plaster casts (obs 2)	0.9969894
	CBCT sagittal views vs. plaster casts (obs 2)	0.9968515

CBCT = cone beam computed tomography; ICC = intra-class correlation coefficient; obs = observer.

**Table 2:** Method effects adjusted for observer. Results from the linear regression mixed model.

Method Effect			
	Mean	95% Confidence Interval	*p* Value
CBCT sagittal views vs. axial views	0.009	−0.021 to 0.040	0.551
CBCT axial views vs. plaster casts	0.176	0.136 to 0.217	<0.001
CBCT sagittal views vs. plaster casts	0.186	0.145 to 0.226	<0.001

CBCT = cone beam computed tomography.

## Data Availability

Data supporting the findings of this study are available upon request from the corresponding author (L.V.).
